# Wet-Spinning Technology for Plant-Based Meat Alternative: Influence of Protein Composition on Physicochemical and Textural Properties

**DOI:** 10.3390/foods14223913

**Published:** 2025-11-15

**Authors:** Swati Kumari, So-Hee Kim, Chan-Jin Kim, Young-Hwa Hwang, Seon-Tea Joo

**Affiliations:** 1 Division of Applied Life Science (BK21 Four), Gyeongsang National University, Jinju 52828, Republic of Korea; swatikumari0724@gmail.com (S.K.);; 2 Institute of Agriculture & Life Science, Gyeongsang National University, Jinju 52828, Republic of Korea

**Keywords:** plant proteins, food quality, bottom-up approach, fibrous-structured meat, meat alternative

## Abstract

The development of a fibrous-structured meat alternative that can perfectly mimic the tribology of the meat is considered to be extremely challenging. In this study, a bottom-up technique, wet spinning, was used to produce a fiber-like structure similar to muscle fiber. Different protein concentrations (0% to 16%) of wheat protein, pea protein isolates, and sodium alginate (2%) were used as an emulsifier and compared with the conventional meat (*longissimus dorsi* muscle) from a barrow in terms of physicochemical (pH, color, moisture content, cooking loss), textural (Texture profile and Warner–Bratzler Shear Force), and sensory parameters. The results from the study showed that the ratio of protein concentration significantly affected the solution behavior, leading to change in the spinnability of solution. The combined protein formulations displayed by a greater range of physicochemical and textural properties, especially hardness and WBSF, ranged from 22 N to 32.20 N and 4.26 to 4.71 kg/cm^2^ in comparison to each other (*p* < 0.05). However, principal component analysis has shown that the overall profiling was significantly different than that of conventional meat (*p* < 0.05). The overall results suggested that the blend of wheat protein and pea protein isolate shows great potential for preparing a variety of structured meat alternatives by optimizing the concentration based on the desired product profiling.

## 1. Introduction

Traditional meat production requires a lot of resources and has a major impact on land degradation, water use, and greenhouse gas emissions. Therefore, sustainable substitutes that can offer comparable nutritional and sensory qualities without putting an environmental load are urgently required. Simultaneously, the increasing population of the world and a sudden shift in the lifestyle, especially food habits, after the COVID-19 outbreak have evoked a great interest in the research regarding the development of products (meat alternatives) that can mimic the tribology and nutritional profiling of meat [1,2,3,4,5]. The development of meat alternatives (MA) is mainly based on two technical approaches: the bottom-up approach (wet spinning, electrospinning, cell culturing, and 3-D printing) and the top-down approach (extrusion, freeze structuring and shear cell) [6]. The bottom-up approach involves manufacturing individual structural elements and assembling them into a larger product, whereas the top-down approach imitates only the larger-scale structure. Among all the top-down techniques, extrusion especially faces hindrances like the use of high temperatures which destroy the nutrients and increase the cost of production, along with complications in operation and instability of the product [7]. Currently, most of the marketed products that are developed through the use of extrusion through thermomechanical processing [8] are burger patties and nuggets, which differ in physical, nutritional, and organoleptic properties from conventional meat, creating a need for conventional imitation meat alternatives [9,10].

The texture of the meat depends upon the arrangement, amount of fat and collagen, along with the orientation, shape, size, and density of the myofibers [11,12,13,14,15,16]. To accurately mimic the textural profile of meat, the primary goal is to develop a component that resembles a muscle fiber through the use of the bottom-up approach, either through wet spinning, 3D-printing, or electrospinning [9,17,18,19,20,21,22]. The wet-spinning technique is used to produce fiber in the textile industry, where the polymers are dissolved in the solvent and subjected to extrusion in the coagulation bath of a non-solvent or chemical required for the solidification of the fibers like rayon, acrylic fibers, aramid, and spandex [23,24,25]. This technique is industrially compatible, with no additional use of any non-food grade component during solution preparation [26].

Previous studies have extensively explored structuring methods such as extrusion and freezing [27,28,29,30,31,32,33,34,35], but the wet-spinning approach remains underutilized, particularly through a lack of understanding how it influences the physicochemical and tribological qualities essential for consumer acceptance of plant-based meat alternatives. [19,36,37]. The studies on wet spinning primarily explored intermolecular interactions, thermal properties, and phase behavior, but not the quality characteristics of the food [38,39,40]. In contrast to pre-existing research, this study aims to apply wet spinning to a novel plant-protein formulation combining pea protein isolate (PPI) and wheat protein (WP) with sodium alginate, evaluating not only the zeta potential and phase behavior but also physicochemical parameters, as well as the textural (texture profile anlaysis and Warner Bratzler Shear Force) and sensory properties in comparison to conventional meat.

## 2. Materials and Methods

### 2.1. Maaterials

Pea protein isolate (PPI) and wheat protein (WP) were purchased from an online platform. The sodium alginate (SA) was acquired from the online market (ESfood, Gunpo-si, Republic of Korea). Calcium chloride was purchased from Qingdao Soda Ash Industrial Development (Qingdao, China). All the materials used for experiments were food grade and were utilized as obtained. For CM (conventional meat), the longissimus dorsi muscle was used from a barrow (Landrace × Yorkshire × Duroc, LYD). Muscle samples from a barrow (6 months old, carcass weight 89 kg) were obtained from a farm in Korea.

### 2.2. Method of Preparation

Different concentrations of WP and PPI sub solutions and SA (2%) were dissolved in distilled water with continuous stirring at room temperature, as mentioned in Table 1. All the solutions were stored in the refrigerator at 4 °C for 12 h. The WP and PPI sub solutions were mixed by equal mass to obtain a protein solution, which was further mixed with SA in a 1:1 ratio (*w*/*w*) to prepare the final spinning solution. Ultimately the solution was subjected to degassing at 20 kHz and 400 W by a Vibra-Cell Ultrasonic Liquid Processor (US/VCX750, Sonics & Materials, Inc. Newtown, CT, USA). The ultrasonic homogenizer probe was immersed about 2 cm with a pulsing cycle of 2 s on and 2 s off for 20 min.

### 2.3. Preparation of Wet-Spun Meat Alternatives

**Figure 1:**
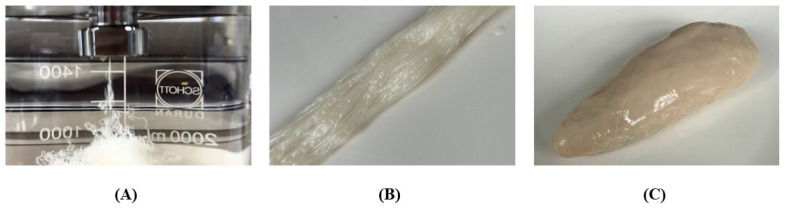
The Image represents the different steps during the wet-spinning technique as (**A**) extrusion of wet-spun solution into the coagulation bath, (**B**) collection of fibers in the form of bundles, and (**C**) image of the wet-spun meat alternative.

The wet-spinning method was used to prepare the imitated fibers (IMFs) from the wet spun solutions (WSSs). The WSSs were extruded by the compression pump through a spinneret of 0.13 mm diameter into the 3% calcium chloride (*w*/*w*) bath and the obtained IMFs were suspended in the coagulation bath for 2 min to attain the complete gelation of the solution, shown in Figure 1A. After that, the IMFs were washed with distilled water at room temperature (20–25 °C) to remove the excess calcium chloride from theIMFs surface, Figure 1B. Finally, the IMFs were collected in Figure 1C to create wet-spun meat alternative (WSMA) and stored for 24 h further analysis.

#### 2.3.1. Zeta Potential

The zeta potential (ZP) testing was performed to determine the stability of the WSSs. The zeta potential was measured with a Zetasizer (Nano-ZS90, Malvern Panalytical, Worcestershire, UK) based on the principle of the dynamic light scattering technique. After preparation, samples were diluted up to 100 times, and then the test was carried out at room temperature in triplicate. The final results were recorded as the mean value.

#### 2.3.2. Solution Analysis

The morphology of WSSs was examined using a confocal laser scanning microscope (Nikon, AX R Nikon Corporation, Tokyo, Japan) using the Nile blue in order to visualize the protein distribution [41,42]. Firstly, the Nile blue solution (0.2% *w*/*v*) was prepared by dissolving 10 mg Nile blue A powder in 5 mL distilled water. Then, 100 μL of staining solution was added to a 5 g solution (solution of PPI & WG), followed by stirring and holding for 3.5 h to mark the protein. Afterward, the stained solution was mixed with the SA solution, which was followed by rapid mixing for 1 min. The WSSs were then diluted 50 times. Then the stained sample was loaded on a microscopic slide and covered by a cover slip, and the image was taken by confocal laser scanning microscopy (CLSM).

#### 2.3.3. pH

The samples’ pH was measured using a digital pH meter (A211 pH Meter, Thermo Fisher Scientific, Waltham, MA, USA). A sample weighing 3 ± 0.05 g was prepared by homogenizing it with 27 milliliters of distilled water (IKA T25, ULTRA-TURAX, Staufen, Germany). The probe was calibrated with the calibration solution at 4.01, 7.00, and 9.99 at 25 °C before the analysis.

#### 2.3.4. Color

A chromometer (CR-300, Konica Minolta, Tokyo, Japan) was used to measure the color indicating the quality and freshness of samples, including CIE L* (lightness), CIE a* (redness), and CIE b* (yellowness), were determined in pentaplicate for each sample from center and the edges. Before analysis the chromometer was equalized with a white plate (Y: 93.5, X: 0.3132: y: 0.3198. The results were expressed as mean ± standard deviation (SD).

#### 2.3.5. Cooking Loss

The cooking loss was used to measure the water holding ability of the samples. 25 ± 0.05 g of the samples were weighed and subjected to the cooking in a water bath at 75 °C for half an hour. The final weight of the samples was measured after 10 min. Equation (1) was used to calculate the cooking loss:

(1) $$CL\%=\frac{\left(W1-W2\right)}{W1}\times 100$$

*CL* = Cooking Loss
*W*1 = Weight of the uncooked samples (g)
*W*2 = Weight of the cooked sample (g)

#### 2.3.6. Moisture Content

The moisture analysis of the samples was performed by AOAC 920 using the Association of Official Agricultural Chemists (AOAC) oven drying methods. 2 ± 0.05 g of samples were weighed into the aluminum dish and were allowed to dry for 16 h at 105 °C in an air dryer (BioFree, BF-150C, Bucheon, Republic of Korea). And then, the aluminum dish was weighed again. The moisture content was calculated as the ratio of the wet and dry weight.

#### 2.3.7. Texture Profile Analysis (TPA)

The TPA was performed by utilizing a texture analyzer (AMETEK). Fresh samples were prepared by cutting them into 1 cm^3^ and placed in the cell. The tests were conducted two times at a maximum load of 180 kg and fixed speed of 100 mm/min. The graph recorded each sample’s hardness, cohesiveness, chewiness, and springiness by the instrument software. All the data were expressed as the mean and standard error of the mean (SEM) for the values measured five times.

#### 2.3.8. Warner–Bratzler Shear Force

The Warner–Bratzler shear force (WBSF) of the fresh samples was measured through the texture analyzer (AMETEK, Berwyn, IL, USA) with a V shaped blade at an angle of 60° to determine the tenderness. The analysis was performed at a speed of 100 mm/min with a load capacity of 50 kg. An approximately 2 cm^2^ cross sectional area was cut from the sample for the purpose of evaluating the force. The data were expressed as the mean and standard deviation (SD) of the values measured five times.

#### 2.3.9. Sensory Analysis

The sensory profile of the samples was analyzed using an electronic sensing system (ETS; INSERT SA402B, Insent, Tokyo, Japan) [43]. The ETS consists of five taste sensors, CA0, C00, AAE, and CT0, responsible for identifying sourness, bitterness, umami, and saltiness. The sensors were stabilized using a reference solution (30 Mm potassium chloride and 0.3 Mm tartaric acid), while the SMT solution containing 0.01% lactic acid, 0.25% monosodium glutamate, and 0.0005% quinine hydrochloride was used for the standardization of membranes. The solutions were prepared by mixing 100 g samples with 400 mL DW at 100 °C for 30 min. The solutions were then centrifuged at 1000× *g* 15 min and then filtered using No. 1 filter paper (Whatman Ltd., Little Chalfont Buckinghamshire, UK). The 80 mL of the supernatant was collected and stored at −70 °C for further analysis.

#### 2.3.10. Statistical Analysis

The statistical analysis was conducted GraphPad Prism (10.1.2; GraphPad, San Diego, CA, USA). All data were represented as mean and SD, and texture profile analysis (TPA) was shown as mean and SEM. The resulting data were analyzed by one-way analysis of variance (ANOVA; Brown–Forsythe and Welch) with the Dunnett T3 test. Analysis of TPA data was performed by one-way ANOVA with Tukey’s multiple comparison test. Principal component analysis (PCA) was conducted to assess the variation in overall analysis among the groups. A score plot was illustrated for the differences in distribution across samples. Statistical significance was determined as *p*-value (*p* < 0.05). All experiments were performed in triplicate (*n* = 3), unless otherwise specified.

## 3. Results and Discussion

### 3.1. Zeta Potential and Phase Behavior

**Figure 2:**
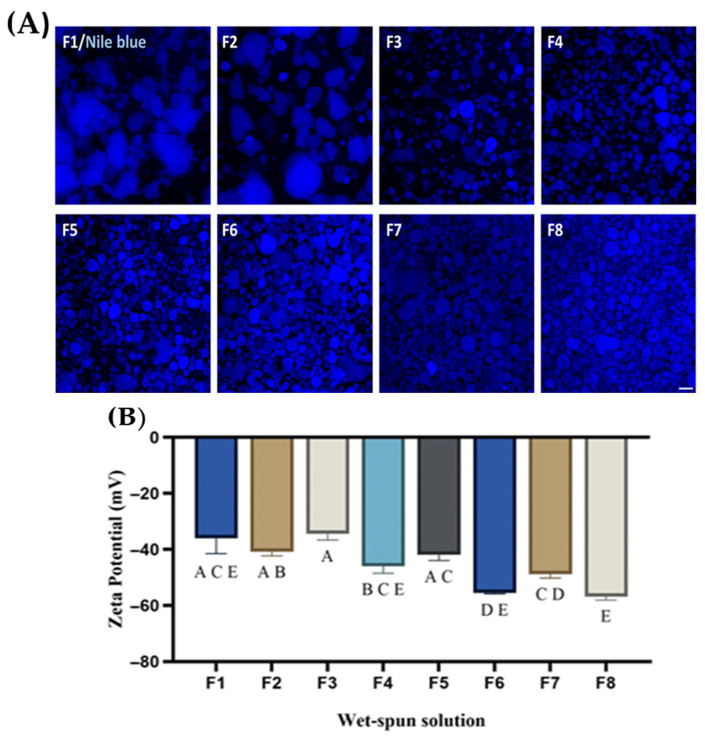
(**A**). The representative fluorescence image of the wet spun solution at different protein concentrations with Nile blue staining; CSLM (confocal laser scanning microscopy. Scale bar = 200 µm; (**B**) Effect of different protein ratios on the zeta potential of the wet spun solution at 2% sodium alginate. ^A–E^ Different letters within a row indicate statistically significant differences between formulations at *p* < 0.05. F1, 16% wheat protein; F2, 14% wheat protein, 4% pea protein isolate; F3: 12% wheat protein, 6% pea protein isolate; F4, 10% wheat protein, 8% pea protein isolate; F5, 8% wheat protein, 10% pea protein isolate; F6, 6% wheat protein, 12% pea protein isolate; F7, 4% wheat protein, 14% pea protein isolate; F8, 16% pea protein isolate.

Figure 2A showed the appearance of a series of WSSs with different protein ratios of PPI and WP after homogenization, dyed with Nile Blue for clear observation to stain the protein. The CLSM images have shown that varying the protein ratio controls the phase separation, which affects the consistency and uniformity of the solution. The F1 and F2 images show that the aggregation of protein (large protein globules) suspended irregularly can be attributed to the aggregation of protein near the isoelectric point of the WP, as supported by the pH in Table 2 [44,45]. This phase behavior is also supported by the low zeta potential of the formulation from Figure 2B (−33.6 mV and −36.7 mV, respectively), displaying weaker electrostatic repulsion and a higher tendency to aggregate [46]. While increasing the concentration of PPI (F3-F8) in the solution seemed to increase the uniformity of the solution, with a progressive size reduction with an increase in the pH as increasing the pH in the alkaline range increases the solubility of the protein, as also observed by [38,47]. Correspondingly, the zeta potential of WSSs reaches up to −57.2 mV for F6, showing increased surface charge and stabilization of electrostatic charges of the solution.

Taken together, the CSLM and zeta potential suggested that increasing the concentration of PPI improves the solution stability by reducing the phase separation and by improving the electrostatic repulsion of the proteins in the solution due to the nature of the PPI, causing the deprotonated and negative charge dominance caused by the presence of glutamic, aspartic and other acidic residue [48,49].

### 3.2. pH and Color

The pH and the color parameters of WSMAs were notably different (*p* < 0.05), asshown in Table 2. The F1 had the lowest pH (5.49), while the other formulations had a higher pH (5.91–6.26) than the CM (5.62). The difference between the CM and WSMAs is due to the difference in the initial pH of PPI and WP. A clear increasing trend was observed from F1 to F8, due to a progressive increase in the concentration of PPI [22]. On the other hand, in the color profile L* showed a significant difference (*p* < 0.05) between CM and WSMAs. The CIE L* showed a clear increase in lightness with increases in the PPI; this may be due to the inherent lighter coloration of pea protein compared to the slightly darker hue of wheat protein, in addition to the increase in the moisture content [50]. The a* values, representing redness, showed more complex fluctuations. Notably, the highest redness was observed in CM (7.17) and F8 (2.28), followed by F5 (1.59) and F3 (1.51), whereas F2 and F7 recorded the lowest a* values (0.55 and 0.57, respectively). These variations could result from differential protein–pigment interactions or the influence of processing conditions, Maillard reactions, which are known to alter red-brown chromophores based on amino acid composition [51,52]. The b* values, corresponding to yellowness, ranged from 14.25 in F2 to 19.29 in F8, showing an overall increasing tendency with higher PP content, while CM has the least (2.08) due to the absence of yellow hue. The rise in yellowness of WSMAs may be associated with the natural pigment composition of pea protein or the influence of processing and protein unfolding, which exposes chromophoric groups.

Altogether, the color analysis emphasizes that pea protein contributes to a lighter, more yellowish, and slightly redder appearance, which could improve the visual appeal of meat alternative fibers or other plant-based food applications.

### 3.3. Cooking Loss and Moisture Content

**Figure 3:**
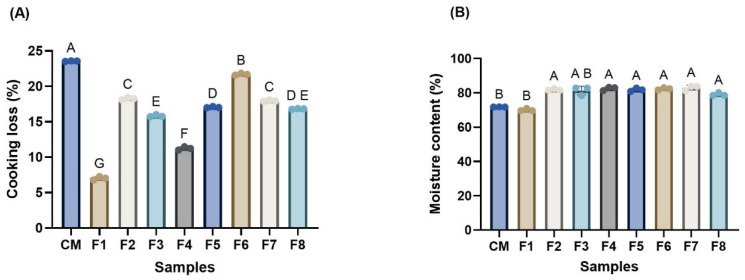
Effect of different protein ratios of wet-spun meat alternative (WSMA) on the cooking loss (**A**) and moisture content (**B**) in comparison to conventional meat. ^A–G^ Different letters indicate statistically significant differences between formulations at *p* < 0.05. CM, conventional meat; F1, 16% wheat protein; F2, 14% wheat protein, 4% pea protein isolate; F3, 12% wheat protein, 6% pea protein isolate; F4, 10% wheat protein, 8% pea protein isolate; F5, 8% wheat protein, 10% pea protein isolate; F6, 6% wheat protein, 12% pea protein isolate; F7, 4% wheat protein, 14% pea protein isolate; F8, 16% pea protein isolate.

The cooking loss (CL) and moisture content (MC) of the WSMAs (F1–F8) showed a subsequent variation (*p* < 0.05) with reference to conventional meat (CM) shown in Figure 3. The F1 exhibited the lowest cooking loss (7.05%), while the CM had the highest CL, around 23.57%. This could be due to the higher concentration of WP, which likely enhanced the water-holding capacity by increasing the stability of the gel network, leading to better water-holding capacity [53,54] and water evaporation and denaturation of protein, leading to the shrinkage of muscle tissues and connective tissues, respectively [55]. Meanwhile, the other WSMAs showed a moderate cooking loss, suggesting the importance of an intermediate balance of PPI and WP. In terms of moisture content, samples from F2 to F7 maintained higher moisture (81.86 to 83.06%) compared to F1 and CM. This enhancement is likely due to the hydrophilic nature of PPI, which contains polar amino acid residues capable of retaining water within the protein matrix [56]. However, in F8, which was formulated exclusively with PPI, the moisture content declined to 79.11%. This reduction may be due to protein aggregation or the absence of a gluten matrix, which could result in a less cohesive structure and uneven water distribution. 

Overall, the data underscore the complementary roles of WP and PPI in modulating cooking loss and moisture content. While WP contributes to structural integrity and water entrapment through gluten network formation, PPI enhances moisture retention through its hydrophilic characteristics [57,58]. An optimal combination of these proteins can therefore reduce the cooking loss and improve water retention, contributing to the desired juiciness and texture in plant-based meat analogs.

### 3.4. Comparative Analysis of WBSF and TPA

The WBSF analysis used to measure the tenderness of the WSMAs revealed a significant difference (*p* < 0.05) in the tenderness regarding CM. Primarily, F1 showed the highest value of WBSF, depicting a firm and tougher fiber arrangement, while increasing the PPI concentration displayed a progressive reduction in WBSF, followed by the lowest WBSF in F8, as shown in Table 3. The obtained results have underlined the critical role of protein type, especially WP, consisting of gluten (gliadin and glutenin), in stabilizing a viscoelastic network and increasing the structural integrity or rigidity and a firm matrix that is resistant to the mechanical stress [59]. On the other hand, the component PPI fails to form a network matrix and also exhibits weaker gelation characteristics, making the products a bit softer and less cohesive in terms of tenderness, making it easily sheared [60].

The TPA hardness of the food is simply the force required during the first bite compression. The WSMAs have significant differences (*p* < 0.05) from CM (25.88). The F4 has the minimum hardness (22 N) in compression compared to the others, including CM; however, the F8 has the maximum hardness (32.20 N). There is no linear trend shown in terms of increasing hardness. The comparatively higher hardness of the F1, F7, and F8 suggested enhanced structural integrity, likely due to stronger protein interaction because of the presence of single proteins [61]. However, the mixed WSMAs have displayed low hardness caused by the disruption of the matrix due to two different proteins’ involvement, as their inability to interact due to difference in bond formation, e.g., PPI forms hydrogen and electrostatic bonds while the WP requires covalent bonding, leads to an antagonistic effect on the hardness. The springiness, gumminess and chewiness have also shown much higher values than CM. Additionally, an increasing trend, as we have seen in the PPI, makes the structures tougher and denser. The cohesiveness of the product has remained stable or shown a slight increase. The reason behind this could be the protein–protein interaction to maintain the internal binding of the structure [62,63].

### 3.5. Comparative Analysis of Taste Characteristics

**Figure 4:**
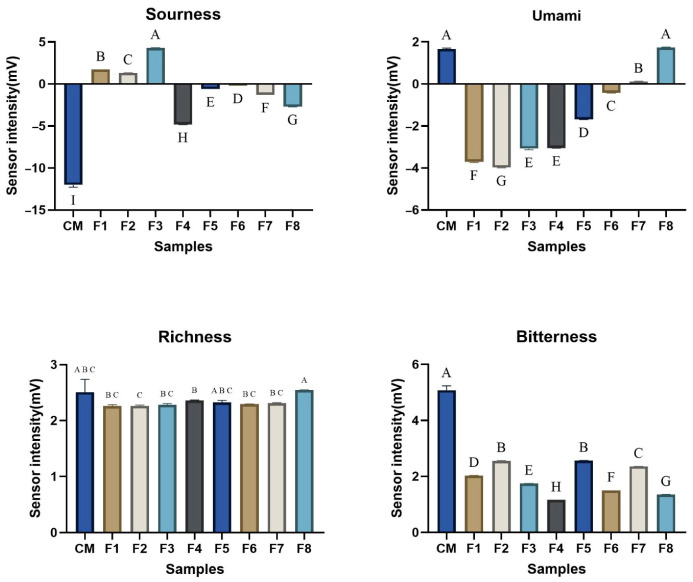
Effect of different protein ratios of wet-spun meat alternatives (WSMAs) on the taste characteristics in comparison to conventional meat. ^A–H^ Different letters within a row indicate statistically significant differences between formulations at *p* < 0.05. CM, conventional meat; F1, 16% wheat protein; F2, 14% wheat protein, 4% pea protein isolate; F3, 12% wheat protein, 6% pea protein isolate; F4, 10% wheat protein, 8% pea protein isolate; F5, 8% wheat protein, 10% pea protein isolate; F6, 6% wheat protein, 12% pea protein isolate; F7, 4% wheat protein, 14% pea protein isolate; F8, 16% pea protein isolate.

The electronic tongue analysis has measured the various parameters (umami, sourness, richness and bitterness) related to the taste characteristics displayed in Figure 4. The results have shown a significant difference (*p* < 0.05). CM has displayed a significant difference in sourness, richness and bitterness in comparison to WSMAs; however, the F8 WSMA showed a similar umami as CM. It suggested that an increase in umami intensity was observed with an increase in PPI concentration, probably due to the high glutamic acid content, making it an important factor for the umami characteristics of the food [64,65]. While the glutamic acid in wheat protein is incorporated into long, folded protein chains, making it unavailable for the taste receptors to sense it [66]. In terms of sourness, the WSMAs, with a high amount of WP, have sourness in positive ranges that may be due to the presence of acidic side chains and a low buffering effect. The sourness peak in F3 may be due to the optimal balance between the acidic residual from PPI and the WP’s buffering ability, which leads to the release of H+ and increases the sourness. Meanwhile, F5–F8 showed a reduction in sourness due to the alkalinity of PPI. The result of the sourness is also supported by the pH result, as the sourness is inversely proportional to pH [67].

The richness is mainly associated with the mouth-coating and flavor complexity sensation in the mouth. A comparable trend has been observed in reference to CM. This richness can be due to an overall increase in the glutamic acid, branched-chain amino acids and hydrophobic peptides, along with the PPI emulsifying ability and fat mimicking behavior and uniformity of the solution and supporting an improved richness [68,69]. On the other hand, bitterness showed a more complex trend, highest in F2 and F5 in correspondence to CM. These results suggested that the bitterness may be related to the specific peptide interaction, especially hydrophobic and sulfur-containing amino acids, which have contributed to bitter flavor when hydrolyzed [70,71]. While the formulation with single protein types F1 and F8 showing lower bitterness may be due to the dominant nature of the protein and limited interaction.

### 3.6. Principal Component Analysis for Quality Analysis

**Figure 5:**
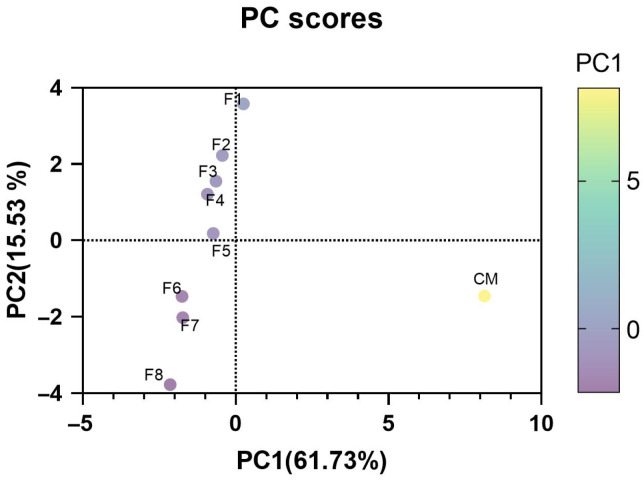
Principal component analysis (PCA score) of quality characteristics of wetspun meat alternative with different protein ratios in comparison to conventional meat. CM, conventional meat; F1, 16% wheat protein; F2, 14% wheat protein, 4% pea protein isolate; F3, 12% wheat protein, 6% pea protein isolate; F4, 10% wheat protein, 8% pea protein isolate; F5, 8% wheat protein, 10% pea protein isolate; F6, 6% wheat protein, 12% pea protein isolate; F7, 4% wheat protein, 14% pea protein isolate; F8, 16% pea protein isolate.

PCA was conducted to analyze the variations in the quality of the WSMAs (pH, color, cooking loss, moisture content, TPA, WBSF, and taste characteristics) in reference to the CM. The PC1 accounted for 61.73% and PC2 15.53% of the total variance in Figure 5. Both components explained approximately 77.26% of the total variance, displaying the difference between them. The color gradient indicates the PCA score value, with yellow hue displaying the higher score while the lighter shades indicate the lower values. The PCA score plot revealed a significant difference with CM clustering separately, indicating the difference in physicochemical and tribological properties. The WSMAs are positioned along PC1, suggesting the changes in the properties based on protein ratio. However, the WSMA may have the potential to mimic the profile of CM, supporting the claim that the optimization of PPI and WP can create resemblance to the CM.

## 4. Conclusions

The objective of this study was to evaluate the influence of wheat protein and pea protein isolate on the physicochemical, textural, and sensory properties of wet-spun meat alternatives in comparison to conventional meat. It was found that the synergistic effect of the proteins has a significant effect on the solution morphology and also the quality characteristics (physicochemical, textural, and sensory properties) of the WSMAs. Findings indicate that whereas the other parameters do not consistently exhibit a linear relationship, the addition of PPI tends to have a linear effect on pH, CIE L* and CIE b* values. However, in comparison to conventional meat, none of the WSMAs have shown the complete imitation of the meat parameters. The study has concluded that the use of the wet-spinning technique can be a promising approach, while optimizing proteins is necessary for the development of a plant-based meat alternative that can imitate the conventional meat. However, no rheological analysis other than viscosity, nutrition, tribometer measurement, and human sensory panel analysis, along with the absence of fat, were certain limitation of this study. These parameters should be included in the future research to allow them to completely mimic the meat’s functionality.

## Figures and Tables

**Table 1:** Concentration of different formulations (*w*/*w*) of the wet-spun solution.

	Wheat Protein (%)	Pea Protein Isolate (%)	Sodium Alginate (%)
F1	16	0	2
F2	14	4	2
F3	12	6	2
F4	10	8	2
F5	8	10	2
F6	6	12	2
F7	4	14	2
F8	0	16	2

**Table 2:** Effect of different protein ratios on the pH and the color (CIE L*, CIE a*, CIE b*) of the wet-spun meat alternative (WSMA) in comparison to conventional meat (CM).

	CM	F1	F2	F3	F4	F5	F6	F7	F8	SEM	*p*-Value
pH	5.62 ^G^	5.49 ^G^	5.91 ^F^	5.98 ^E^	6.05 ^D^	6.07 ^D^	6.14 ^C^	6.20 ^B^	6.26 ^A^	0.007	<0.001
Color											
CIE L*	53.79 ^I^	62.88 ^H^	64.96 ^G^	66.26 ^F^	68.50 ^E^	70.45 ^D^	71.45 ^C^	72.46 ^B^	73.90 ^A^	0.049	<0.001
CIE a*	7.17 ^A^	1.295 ^E^	0.55 ^F^	1.51 ^D^	1.49 ^D^	1.59 ^C^	1.24 ^E^	0.57 ^F^	2.28 ^B^	0.008	<0.001
CIE b*	2.08 ^F^	16.89 ^B^	14.25 ^E^	16.05 ^D^	16.22 ^C,D^	16.41 ^C^	15.94 ^D^	17.11 ^B^	19.29 ^A^	0.008	<0.001

Values are presented as mean ± pooled standard error of the mean. Different superscript letters ^A–I^ within the same row indicate significant differences (*p* < 0.05). CM, conventional meat; F1, 16% wheat protein; F2, 14% wheat protein, 4% pea protein isolate; F3, 12% wheat protein, 6% pea protein isolate; F4, 10% wheat protein, 8% pea protein isolate; F5, 8% wheat protein, 10% pea protein isolate; F6, 6% wheat protein, 12% pea protein isolate; F7, 4% wheat protein, 14% pea protein isolate; F8, 16% pea protein isolate.

**Table 3:** Effect of different protein ratios on the wet-spun meat alternative (WSMA) and texture profile analysis of the wet-spun meat alternatives in comparison to conventional meat (CM).

	CM	F1	F2	F3	F4	F5	F6	F7	F8	SEM	*p*-Value
WBSF (kg/cm^2^)	1.22 ^D^	4.71 ^A^	4.62 ^A^	4.57 ^A^	4.51 ^A^	4.45 ^B^	4.40 ^B^	4.34 ^B,C^	4.26 ^C^	0.007	<0.0001
Hardness (N)	25.88 ^B,C^	26.53 ^B^	25.00 ^D^	23.03 ^E^	22.00 ^F^	23.53 ^E^	25.03 ^C,D^	27.02 ^B^	32.20 ^A^	1.990	<0.0001
Springiness	0.86 ^F^	0.87 ^F^	0.87 ^F^	0.88 ^E^	0.88 ^E^	0.89 ^D^	0.91 ^C^	0.92 ^B^	0.93 ^A^	0.049	<0.0001
Gumminess (N)	3.63 ^H^	6.50 ^G^	6.97 ^F,G^	7.80 ^E,F^	8.20 ^E^	9.00 ^D^	10.50 ^C^	12.00 ^B^	13.27 ^A^	0.068	<0.0001
Chewiness (N)	4.79 ^H^	6.20 ^G^	6.80 ^F^	7.20 ^E,F^	7.50 ^E^	8.10 ^D^	8.91 ^C^	9.50 ^B^	11.73 ^A^	0.008	<0.0001
Cohesiveness	0.26 ^C,D,E,F^	0.25 ^F^	0.25 ^F^	0.26 ^E^	0.26 ^E^	0.27 ^D^	0.28 ^C^	0.29 ^B^	0.30 ^A^	0.008	<0.0001

^A–H^ Different letters within a row indicate statistically significant differences between formulations at *p* < 0.05. CM, conventional meat; F1, 16% wheat protein; F2, 14% wheat protein, 4% pea protein isolate; F3: 12% wheat protein, 6% pea protein isolate; F4, 10% wheat protein, 8% pea protein isolate; F5, 8% wheat protein, 10% pea protein isolate; F6, 6% wheat protein, 12% pea protein isolate; F7, 4% wheat protein, 14% pea protein isolate; F8, 16% pea protein isolate.

## Data Availability

The original contributions presented in this study are included in the article. Further inquiries can be directed to the corresponding author.
